# Tyrotoxicon—Cheese Poison

**Published:** 1885-08

**Authors:** V. C. Vaughan


### Tyrotoxicon—Cheese Poison.

Abstract of a Paper by Prof. V. C. Vaughan, M. D., Ph. D.

At the meeting of the Michigan State Board of Health, July 14, 1885, Dr. Vaughan presented a report of his investigations on poisonous cheese. It is well known that cases of severe illness follow the eating of some cheese. Such instances are of frequent occurrence in the North German countries and in the United States. In England, they are less frequently observed; while in France, where much cheese is made and eaten, these cases are said to occur very rarely. A few years ago, the reputation of a large cheese factory in Northern Ohio was destroyed by the great number of cases of alarming illness arising from eating its cheese. Dairymen know this cheese as "sick" cheese.

### KINDS OF CHEESE THAT ARE POISONOUS.

A German author says: "The numerous kinds of soft cheese, prepared in small families, or on small farms, are generally the cause of the symptoms; while it is quite exceptional to hear of symptoms arising from the use of cheese prepared in large quantities." Some two years ago, a family in Alpena, Mich., was poisoned by eating of cottage cheese; but the cheese which poisoned so many in this State last year was made at one of the largest factories in the State, and by a thoroughly experienced cheese-maker. The old foul-smelling cheese, such as Limburger and Schweitzer, have never been known to be poisonous.

### EFFECTS OF THE CHEESE.

The symptoms produced by "sick" cheese, as reported by German and American physicians, agree quite closely, and are as follows: Dryness of the mouth and throat, with a sense of constriction, nausea, vomiting, diarrhœa, headache, sometimes double vision, and marked nervous prostration. In rare instances, the sufferer dies from collapse. As a rule, recovery occurs in a few hours, or, at most, after a few days. The symptoms of cheese-poisoning and those of sausage, canned meats and fish-poisoning are very similar; though death results more frequently from the others mentioned than from cheese-poisoning.

### APPEARANCE OF THE CHEESE.

The samples of cheese examined had no peculiarities of appearance, odor or tas e, by which it could be distinguished from good cheese. It is true that if two pieces of cheese—one poisonous and the other wholesome—were offered to a dog or a cat, the animal would select the good cheese. But this was probably due to an acuteness of the sense of smell possessed by the animal, and not belonging to man. Indeed, if a person tasted a cheese knowing that it was poisonous, he might detect a sharpness of taste which would not ordinarily be noticed.

HAVE WE ANY READY MEANS OF RECOGNIZING POISONOUS CHEESE?

There is no certain means, aside from a chemical examination, by which a poisonous cheese can be distinguished from a wholesome one. The most reliable ready method is probably that proposed by Dr. Vaughan a year ago, and it is as follows: Press a small strip of blue litmus paper (which can be obtained at any drug store) against a freshly cut surface of the cheese; if the paper is reddened instantly and intensely, the cheese may be regarded with suspicion. When treated in this way, any green cheese will redden the litmus paper, but ordinarily the reddening will be produced slowly, and will be slight. If the piece of cheese be dry, a small bit should be rubbed up with an equal volume of water, and the paper should then be dipped in the water. Dr. Vaughan does not regard the above test as free from error, but as the most reliable ready means now known. Every grocery-man should apply this test to each fresh cheese which he cuts. The depth of the reddening of the paper may be compared with that produced by cheese which is known to be wholesome.

EFFECTS ON THE LOWER ANIMALS.

Dogs and cats, at least, are not affected by eating poisonous cheese. This is probably due to the fact that they do not get enough of the poison from the amount of cheese which they eat. The pure isolated poison in sufficient doses would undoubtedly produce upon the lower animals effects similar to those produced on man.

NATURE OF THE POISON.

Dr. Vaughan has succeeded in isolating the poison, to which he has given the name tyrotoxicon (from two Greek words, which mean cheese and poison). It is a product of slight putrefaction in the cheese, which, probably, occurs in the vat, as the curd has been known to poison a person. By this slight putrefaction, or excessive fermentation, as it may be called, a large amount of butyric acid is formed, and this, in the presence of the casein of the cheese, is capable of developing a poison. Different samples of poisonous cheese contain different amounts of the poison. The same weight of cheese from one cake furnished three times as much poison as that from another cake. The poison was obtained in long, needle-shaped crystals, which are freely soluble in water, chloroform, alcohol and ether. The smallest visible fragment of a crystal placed upon the end of the tongue causes a sharp, stinging pain at the point of application, and, in a few minutes, dryness and constriction of the throat. A slightly larger amount produced nausea, vomiting and diarrhœa. The poison is volatile at the temperature of boiling water, and for this reason even poisonous cheese may be eaten with impunity after being cooked. The substance has also a marked, pungent odor, and through the nose one can obtain sufficient of the volatile poison to produce dryness of the throat. This is true, however, only of the isolated poison. In the cheese, the taste and odor of the poison are both modified to such an extent that they would not be recognized, as has already been stated.

The first step in the study of cheese-poisoning has now been taken, by finding out what the poison is. Efforts will be made to ascertain the means for preventing its formation.

